# HIV-1 cross-resistance to second-generation non-nucleoside reverse transcriptase inhibitors among individuals failing antiretroviral therapy in Cameroon: implications for the use of long-acting treatment regimens in low- and middle-income countries

**DOI:** 10.1093/jacamr/dlaf059

**Published:** 2025-04-28

**Authors:** Davy-Hyacinthe Gouissi Anguechia, Yagai Bouba, Ezechiel Ngoufack Jagni Semengue, Desire Takou, Collins Ambe Chenwi, Vincent Kamaël Mekel, Grace Angong Beloumou, Alex Durand Nka, Aude Christelle Ka’e, Sandrine Claire Ndjeyep Djupsa, Vittorio Colizzi, Nicaise Ndembi, Alexis Ndjolo, Dora Mbanya, Carlo-Federico Perno, Joseph Fokam

**Affiliations:** Virology Laboratory, Chantal BIYA International Reference Centre for Research on HIV/AIDS Prevention and Management, Yaoundé, Cameroon; Faculty of Medicine and Biomedical Sciences, University of Yaoundé I, Yaoundé, Cameroon; Virology Laboratory, Chantal BIYA International Reference Centre for Research on HIV/AIDS Prevention and Management, Yaoundé, Cameroon; Faculty of Medicine and Surgery, Saint Camillus International University of Health Sciences, Rome, Italy; Virology Laboratory, Chantal BIYA International Reference Centre for Research on HIV/AIDS Prevention and Management, Yaoundé, Cameroon; National HIV Drug Resistance Working Group, Ministry of Public Health, Yaoundé, Cameroon; Virology Laboratory, Chantal BIYA International Reference Centre for Research on HIV/AIDS Prevention and Management, Yaoundé, Cameroon; National HIV Drug Resistance Working Group, Ministry of Public Health, Yaoundé, Cameroon; Virology Laboratory, Chantal BIYA International Reference Centre for Research on HIV/AIDS Prevention and Management, Yaoundé, Cameroon; National HIV Drug Resistance Working Group, Ministry of Public Health, Yaoundé, Cameroon; Department of Experimental Medicine, University of Rome ‘Tor Vergata’, Rome, Italy; Virology Laboratory, Chantal BIYA International Reference Centre for Research on HIV/AIDS Prevention and Management, Yaoundé, Cameroon; Department of Experimental Medicine, University of Rome ‘Tor Vergata’, Rome, Italy; Virology Laboratory, Chantal BIYA International Reference Centre for Research on HIV/AIDS Prevention and Management, Yaoundé, Cameroon; Department of Experimental Medicine, University of Rome ‘Tor Vergata’, Rome, Italy; Virology Laboratory, Chantal BIYA International Reference Centre for Research on HIV/AIDS Prevention and Management, Yaoundé, Cameroon; Virology Laboratory, Chantal BIYA International Reference Centre for Research on HIV/AIDS Prevention and Management, Yaoundé, Cameroon; Virology Laboratory, Chantal BIYA International Reference Centre for Research on HIV/AIDS Prevention and Management, Yaoundé, Cameroon; Virology Laboratory, Chantal BIYA International Reference Centre for Research on HIV/AIDS Prevention and Management, Yaoundé, Cameroon; EUROBIOPARK and UNSECO board for Biotechnology, University of Rome Tor Vergata, Rome, Italy; Faculty of Sciences and Technology, Evangelical University of Cameroon, Bandjoun, Cameroon; Africa Centres for Disease Control and Prevention, Addis Ababa, Ethiopia; Institute of Human Virology, University of Maryland, Baltimore, USA; Virology Laboratory, Chantal BIYA International Reference Centre for Research on HIV/AIDS Prevention and Management, Yaoundé, Cameroon; Faculty of Medicine and Biomedical Sciences, University of Yaoundé I, Yaoundé, Cameroon; Faculty of Medicine and Biomedical Sciences, University of Yaoundé I, Yaoundé, Cameroon; Virology Laboratory, Chantal BIYA International Reference Centre for Research on HIV/AIDS Prevention and Management, Yaoundé, Cameroon; Bambino Gesù Children Hospital, IRCCS, Rome, Italy; Virology Laboratory, Chantal BIYA International Reference Centre for Research on HIV/AIDS Prevention and Management, Yaoundé, Cameroon; Faculty of Medicine and Biomedical Sciences, University of Yaoundé I, Yaoundé, Cameroon; National HIV Drug Resistance Working Group, Ministry of Public Health, Yaoundé, Cameroon; Faculty of Health Sciences, University of Buea, Buea, Cameroon; Central Technical Group, National AIDS Control Committee, Yaoundé, Cameroon

## Abstract

**Background:**

Several long-acting antiretroviral treatment regimens contain second-generation non-nucleoside reverse transcriptase inhibitors (2ndGenNNRTI). As first-generation NNRTIs (1stGenNNRTI) exhibit some cross-resistance with 2ndGenNNRTI, we sought to evaluate the rate of acquired cross-resistance to 2ndGenNNRTI and its determinants at treatment failure in a typical low- and middle-income country (LMIC) such as Cameroon.

**Patients and methods:**

A facility-based cross-sectional study was conducted among patients failing first-/second-line regimens between 2019 and 2023 in Cameroon. HIV-1 Sanger sequencing was performed on plasma and resistance-associated mutations (RAMs) to etravirine, rilpivirine and doravirine were interpreted using HIVdb program v.9.5.0 (HIVdb penalty scores were,  ≥60, high resistance; 15–59, intermediate resistance and  <15, susceptible) and the IAS-USA 2022 list.

**Results:**

Overall, 653 individuals previously exposed to 1stGenNNRTI were enrolled [median (IQR) age 39 (26–46) years and viraemia 59 370 (10 442–244 916) copies/mL]. Importantly, 361 participants were on 1stGenNNRTI-based first-line and 292 on protease inhibitor-based second-line regimen. NNRTIs RAMs were found in up to 90.64% of individuals, with 36.45% having more than three RAMs. Concerning 2ndGenNNRTIs, 77.18% of individuals harboured RAMs conferring high or intermediate-level resistance, with the predicted efficacy of etravirine, doravirine and rilpivirine being 47.17%, 33.23% and 32.31%, respectively. Major 2ndGenNNRTIs RAMs were driven by Y181C (23.74%), K101E (8.57%), Y188L (8.42%) and H221Y (8.42%), while minor RAMs were A98G (18.83%), G190A (18.68%) and P225H (14.70%). A higher prevalence of RAMs was observed in those failing first-line versus second line (81.71% versus 71.57%, respectively, *P* < 0.001), driven predominantly by the difference in doravirine-RAMs [first line (72.85%) versus second line (59.58%), *P* < 0.001].

**Conclusions:**

Among patients failing treatment in Cameroon, there is a high-level of cross-resistance to 2ndGenNNRTI due to wide exposure to 1stGenNNRTI. Thus, in LMICs sharing similar programmatic features, the use of NNRTI-sparing regimens should be prioritized as a public health approach, while second-generation-NNRTI long-acting regimens should be guided by genotyping or for clients without previous exposure to NNRTIs.

## Introduction

The use of combination antiretroviral therapy (cART) has led to an unprecedented success in the control of human immunodeficiency virus type 1 (HIV-1) infection, significantly improving the lifespan of people living with HIV (PLHIV). However, the administration of antiretrovirals (ARVs) is still life long.^1^ Currently, major steps of HIV life cycle are targeted by an ARV, with most cART targeting at least two of the three main HIV enzymes, which are reverse transcriptase (RT), integrase (IN) and protease (PR) and all involved in viral replication and serving as key drug targets in the management of PLHIV.^2,3^ Owing to the extensive use of these ARV drug targets, the emergence of HIV drug resistance (HIVDR) has become a challenge to the achievement or maintenance of virological success in several low- and middle-income countries (LMICs).^4^ Even though such poor treatment outcomes are often due to adherence issues, the level of genetic barrier to resistance of ARV drug classes or regimens plays a key role.^5^ Of note, non-nucleoside reverse transcriptase inhibitors (NNRTI) have a lower genetic barrier to resistance when compared to other ARV classes such as integrase strand transfer inhibitors (INSTIs) and ritonavir-boosted protease inhibitors (PI/r).^5^ An alarming level of HIVDR, which is essentially driven by resistance to NNRTIs, was previously reported in Cameroon.^6^ Of note, NNRTIs are drugs targeting the RT enzymes and divided into two distinct generations based essentially on their defined level of barrier to resistance: the first- and second-generation NNRTIs. The first-generation NNRTIs (1stGenNNRTI) include nevirapine (NVP) and efavirenz (EFV), both approved and extensively used for treating HIV infection since the late 1990s.^7^ Globally, the high rate of 1stGenNNRTI resistance led to the recommendation of NNRTIs-sparing regimens as preferred ART in most LMICs, and then to the development of second-generation NNRTI (2ndGenNNRTIs).^8,9^ Of relevance, 2ndGenNNRTIs used for treating HIV infection are etravirine (ETR), Rilpivirine (RPV) and Doravirine (DOR), were respectively approved in 2008, 2011 and 2018, and recommended even for those with previous exposure or failure to 1stGenNNRTIs, if not carrying mutations specific for 2ndGenNNRTIs.^9,10^ Meanwhile cross-resistance from 1stGenNNRTI mutations (common in settings with long-term exposure to 1stGenNNRTIs) may affect efficacy of 2ndGenNNRTIs.^11,12^ It is also important to point out that, among the 2ndGenNNRTIs, RPV has a low genetic barrier to resistance and might not overcome all 1stGenNNRTI mutations as K103N is rarely alone, while DOR has the advantage to overcome Y181C mutant, when present alone.^13^

With the goal to improve the quality of life of PLHIV, long-acting (LA) formulations have been developed, most of which are today based on 2ndGenNNRTIs. For instance, a LA injectable regimen based on a 2ndGenNNRTI (RPV) and INSTI pcabotegravir (CAB) has been recently approved by the US Food and Drug Administration (FDA) for the maintenance of virological suppression in adults and adolescents aged 12 years and above.^9,14^ This regimen has been demonstrated to be safe and non-inferior to traditional three drug-based regimen.^15,16^ Moreover, there are other dual therapy strategies, including RPV/dolutegravir (DTG), which have been validated and recommended for virologically suppressed patients,^17^ DOR/DTG^18,19^ and ETR/raltegravir (RAL),^20^ which are evaluated in pilot studies but not yet recommended, and DOR/Islatravir (ISL), which is still in clinical trials.^21^ Given the potential of LA formulations to overcome challenges related to daily or regular pill intake, stigma and ART adherence, PLHIV are increasingly showing an interest in this new and attractive treatment strategy, including those living in LMICs. The introduction of currently approved LA in LMICs should be done with caution due to the wide and long-term exposure to 1stGenNNRTIs, drugs known to have low genetic barrier to resistance (i.e. a single mutation leading to high-level resistance). This is particularly true for countries such as Cameroon where ∼90% of PLHIV have previous exposure to nevirapine and/or efavirenz either for prevention or as part of ART. Therefore, most (if not all) LMICs may require to set-up adapted strategies to guarantee a successful transition into the LA era. Importantly, one of the eligibility criteria for the FDA-approved CAB + RPV LA is the absence of resistance mutations to CAB and RPV.^9,22^ In this regard, previous findings revealed 12% pre-treatment resistance (PDR) and 80%–90% acquired drug resistance to 1stGenNNRTIs,^23,24^ associated with high risk of virological failure to EFV- or NVP-containing regimens in several LMICs.^5^

Cameroon is a LMIC with a generalized HIV epidemiology (2.7% prevalence) and with the introduction of ART as a public health approach since May 2000. Over 448 818 PLHIV received ART by the end of 2023 (93.3% of those know their HIV status); of them, ∼97% are on first-line and 3% on second-line ART regimens, most being exposed to 1stGenNNRTIs. In this context, the introduction of LA regimens such as CAB + RPV might be convenient, pending investigations to identify eligibility and to determine the most suitable populations for minimizing risks of monofunctional CAB (due to RPV cross-resistance). We have previously reported a baseline analysis of <1% INSTI-resistance (0% in naive individuals and 0.9% among ART-experienced),^25^ mainly explained by the recent introduction of an INSTI-based regimen (Tenofovir/Lamivudine/Dolutegravir) as the preferred first-line regimen in Cameroon, with ∼78.8% receiving Tenofovir/Lamivudine/Dolutegravir at the time of this study.^26^ On the other hand, due to the prolonged exposure to 1stGenNNRTIs among ART-experienced individuals, resistance to these NNRTIs is concerning and might limit the translational application of current LA at country-level and even beyond. So far, none of the 2ndGenNNRTIs are available in Cameroon or in several LMICs due to scarcity of evidence for policy implementation. To provide informed guidance on the use of 2ndGenNNRTI-based regimens containing RPV LA combinations in LMICs, a thorough understanding of NNRTI mutations and their adequacy of cross-resistance to 2ndGenNNRTIs will be a great asset for ART programmes. In this frame, our study objective was to evaluate the burden of HIV-1 resistance mutations to 2ndGenNNRTIs among PLHIV failing ART and the potential efficacy of RPV, ETR and DOR in the Cameroonian context.

## Patients and methods

### Study design

This is a facility-based cross-sectional study conducted among 1stGenNNRTI-exposed patients failing first-/second-line ART regimens eligible for HIVDR testing at the ‘Chantal BIYA’ International Reference Centre (CIRCB) for research on HIV/AIDS prevention and management (CIRCB) in Yaoundé-Cameroon between 2019 and 2023. The CIRCB is the national reference laboratory for HIV genotypic drug resistance monitoring and surveillance in Cameroon and currently a WHO candidate laboratory for HIVDR surveillance. Sample collected from eligible individuals from the treatment centres all over the country are shipped to CIRCB, where resistance tests are performed. For the purpose of this study, individuals failing ART with documented previous exposure to 1stGenNNRTIs (NVP and EFV) for at least 12 months, experiencing virological failure (two consecutive viral load measurements ≥1000 copies/mL) and with HIV-1 sequences genotyped, were enrolled.

### HIV-1 genotypic drug resistance testing

HIV-1 genotyping resistance testing was performed on plasma samples using the Sanger sequencing method. This genotyping assay, which sequences the HIV-1 *pol* gene (PR, 1–99 amino acids; RT, 1–251 amino acids), was performed using the in-house protocol, as previously described.^27^ Briefly, viral RNA was extracted using a commercial kit (QIAmp Viral RNA mini-kit, Qiagen Hilden, Germany). The RNA extracts were also amplified following the developed in-house RT polymerase chain reaction (RT–PCR) protocol. For samples that were insufficiently amplified after the first round of PCR, a nested PCR (semi-nested PCR) was performed. The expected cDNA was ∼1510 bp in length. The amplified products from the pol region were completely sequenced in the sense and antisense orientations using an automated sequencer (ABI 3500 Genetic Analyzer), with seven different overlapping sequence-specific primers.

### Sequence analysis for HIVDR profiling and subtyping

The resulting forward and reverse nucleotide sequence were assembled, and manually edited with SeqScape v.2.7 (Applied Biosystems) or RECall v.2.28. The profile of resistance-associated mutations (RAMs) was identified according to the 2022 International AIDS Society (IAS-USA) major resistance mutation list.^28^ Additionally, RAMs that were not included in IAS-USA RAMs list but obtained a penalty score ≥15 using the Stanford program (HIVdb, v.9.5, updated 22 August 2023) were considered resistance mutations for DOR, RPV and ETR. Drug susceptibility profile was established using three different algorithms for a consensus in determining the predictive efficacy of 2ndGenNNRTIs (ETR, DOR, RPV): Stanford HIVdb v.9.5.0 (https://hivdb.stanford.edu/hivdb/by-patterns/), ANRS algorithm.v33 (National Agency for AIDS Research, https://hivfrenchresistance.org/) and HIV-GRADE (Genotypic Resistance-Algorithm Deutschland, https://www.hiv-grade.de/grade_new/). For the HIVdb algorithm, resistance to NNRTIs was interpreted using the genotypic scoring system for drug susceptibility with the following penalty: ≥60 high resistance; 15–59 intermediate resistance and <15 susceptible. The GRADE HIV-1 tool allows comparison of drug resistance interpretation systems (ANRS, Rega, Stanford) while presenting rules and results of other drug resistance algorithms for a given sequence simultaneously.^29^ Viral subtypes were identified using online bioinformatic tools [REGA subtyping tool v.3.0 (http://dbpartners.stanford.edu:8080/RegaSubtyping/stanford-hiv/typingtool/), COMET-HIV-1 (https://comet.lih.lu/)] and further confirmed by molecular phylogeny using MEGA v.11.

### Data management and statistical analyses

Quality assurance (demographic and sequence data) used for this study was ensured through double checking by two different study investigators. Qualitative variables were presented as number and percentages, while quantitative variables were presented as median and interquartile range (IQR). For the statistical tests, comparisons were performed using Chi*-*square test (*χ*^2^) or Fisher’s exact test as appropriate for categorical variables; and the Mann–Whitney *U*-test or Kruskal–Wallis test as appropriate, for continuous variables. The agreement between ETR, RPV and DOR susceptibility and resistance scores between the Stanford HIV database, ANRS and HIV-GRADE were assessed using the kappa statistic^30^ and Kendall’s tau.^31^ Kendall’s tau ranges were interpreted as follows: a value of 0.2–0.39 indicated a weak positive agreement, 0.40–0.59 a moderate positive agreement and 0.60–0.80 a strong positive agreement.^31^ The significance level was set at *P* < 0.05 for all the tests.

### Ethical considerations

This study was conducted in accordance with the principles of the Declaration of Helsinki and national regulations. The study received an administrative authorization from the Director General of CIRCB (reference no. 1929/020); ethical clearance from the Institutional Ethics Committee of the Faculty of Medicine and Biomedical Sciences of the University of Yaoundé I (reference no. 263/UY1/FMSB/VDRC/DAASR/CSD); informed consent was provided and data confidentiality and privacy were duly respected.

## Results

### Characteristics of the study population

**Figure 1.**
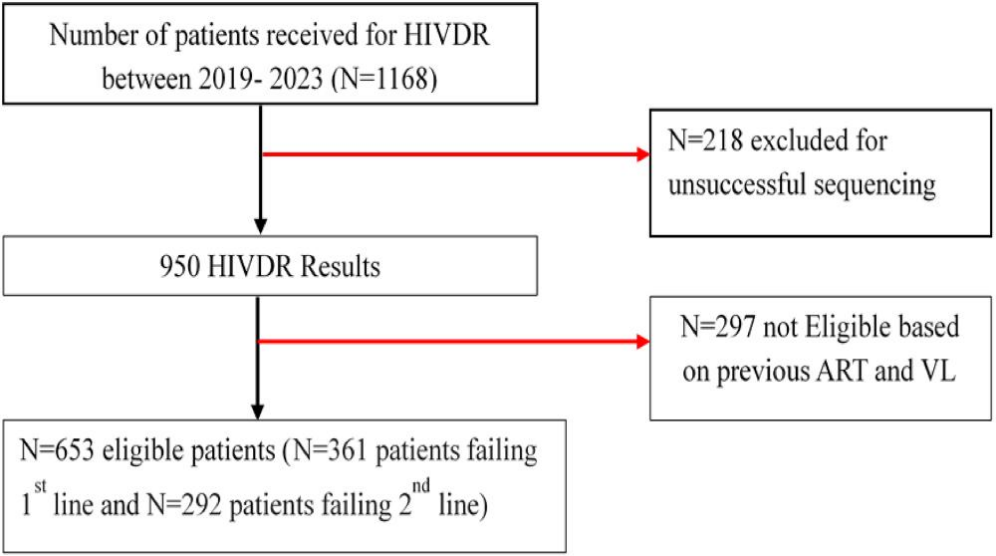
Study flow chart. N, number; VL, Viral load.

A total of 653 individuals were included in this study. Of these, 361 were failing their first-line ART (NNRTI-based regimens) and 292 were failing their second-line ART (PI-based regimens) (Figure 1). The median [IQR] age and viraemia at failure were 39 [26–46] years and 59 370 [10 442–244 916] copies/mL, respectively. Table 1 presents the demographic and clinical characteristics of the study population.

**Table 1.** Demographic, clinical and virological characteristics of individuals

Variables		Frequency (*N* = 653)	Proportion (%)
Gender	Male	412	63.09
	Female	241	36.91
Age categories (years)	Adolescent (10–19)	123	18.84
	Adults (≥20)	530	81.16
Contextual viraemia (copies/mL)	(1000–9999)	160	24.50
	(10 000–99 999)	221	33.84
	≥100 000	272	41.65
ART treatment line	First line	361	55.28
	Second line	292	44.71
History of exposure to 1stGenNNTIs	Efavirenz	402	61.55
	Nevirapine	218	33.39
	Efavirenz and Nevirapine	33	5.06

### HIV-1 genetic diversity

The analysis of HIV-1 PR-RT region revealed a high HIV-1 genetic diversity. All the individuals were infected with HIV-1, group M. Overall, CRF_02AG subtype was the most prevalent, representing 67.23% (439/653). Other detected recombinant and complex forms included CRF01_AE (1.23%), CRF22_01A1 (0.61%), CRF06_cpx (0.46%), CRF09_cpx (0.44%), CRF11_cpx (3.37%), CRF13_cpx (0.46%), CRF18_cpx (1.84%), CRF25_cpx (0.77%) CRF37_cpx (0.77%) and URF (0.15%). About 22.67% (148/653) of individuals harboured a pure HIV-1 subtype, including subtype A (0.31%), A1 (9.04%), B (0.15%), C (0.15%), D (2.91%), F2 (3.83%), G (5.82%) and H (0.46%).

### Prevalence of resistance mutations to second-generation NNRTIs

Overall, the prevalence of individuals with at least one major resistance mutations to NNRTI was 90.64% (592/653). Regarding 2ndGenNNRTI, the proportion of patients harbouring RAMs (conferring high- or intermediate-level resistance; HIVdb score >15) was 77.18% [95%CI: 73.71–80.24]. Major and accessory resistance mutations represented 67.68% (442/653) and 64.93% (424/653), respectively. A percentage of 36.45% (238/653) people were found to have more than three RAMs to NNRTI (Table 2). In addition, we observed that individuals failing their first-line NNRTI-based ART had a significantly higher resistance rate (81.71%), when compared to those failing second-line ART (71.57%), *P* < 0.001.

**Table 2.** RAMs to NNRTIs among individuals failing ART

Resistance mutations	Total (*N* = 653)	Failing first line (*N* = 361, 55.28%)	Failing second line (*N* = 292, 44.72%)	*P* value
Overall resistance rate, *n* (%)	504 (77.18)	295 (81.71)	209 (71.57)	**<0**.**001**
A98G	123 (18.83)	67 (18.55)	56 (19.17)	0.84
L100I	38 (5.81)	31 (8.59)	7 (2.39)	**<0**.**001**
K101P	12 (1.83)	7 (1.93)	5 (1.71)	0.48
K101E	56 (8.57)	28 (7.75)	28 (9.58)	0.40
K103N	313 (47.93)	211 (58.45)	102 (34.93)	**<0**.**001**
E138A	27 (4.13)	16 (4.43)	11 (3.76)	0.67
E138G	5 (0.76)	2 (0.55)	3 (1.03)	0.66
E138K	2 (0.31)	1 (0.27)	1 (0.34)	1.00
E138Q	22 (3.36)	13 (3.59)	9 (3.08)	0.71
V106A	17 (2.60)	13 (3.59)	4 (1.37)	0.02
V106I	48 (6.88)	25 (6.92)	23 (7.87)	0.64
V106M	14 (2.14)	10 (2.77)	4 (1.37)	0.28
V179L	4 (0.61)	3 (0.83)	1 (0.38)	0.63
Y181C	155 (23.74)	92 (25.48)	63 (21.57)	**<0**.**001**
Y181I	1 (0.15)	0 (0.00)	1 (0.34)	0.44
Y181V	8 (1.22)	3 (0.83)	5 (1.71)	0.47
Y188L	55 (8.42)	27 (7.47)	24 (8.22)	0.73
Y318F	2 (0.31)	2 (0.55)	0 (0.00)	0.50
G190A	122 (18.68)	68 (18.83)	54 (18.49)	0.91
G190E	2 (0.31)	1 (0.27)	1 (0.34)	1.00
G190S	12 (1.83)	7 (1.93)	5 (1.71)	0.83
H221Y	55 (8.42)	38 (10.52)	17 (5.82)	**0**.**03**
P225H	96 (14.70)	60 (16.62)	36 (12.32)	0.18
F227L	28 (4.28)	20 (5.54)	8 (2.74)	0.08
M230L	28 (4.28)	16 (4.43)	12 (4.11)	0.84
L234I	15 (2.30)	12 (3.32)	3 (8.76)	0.07
Distribution of NNRTI-RAMs				
0 NNRTI-RAM	69 (10.56)	24 (6.65)	45 (15.41)	**<0**.**001**
1 NNRTI-RAM	50 (7.66)	21 (5.82)	29 (11.11)	**0**.**05**
2 NNRTI-RAMs	129 (19.76)	72 (19.94)	57 (15.78)	0.88
3 NNRTI-RAMs	167 (25.57)	88 (24.38)	79 (27.05)	0.43
>3 NNRTI-RAMs	238 (36.45)	156 (43.21)	82 (28.08)	**<0**.**001**

RAMs were interpreted according to the HIVdb and the IAS-USA 2022 list of mutations.^28^  *P* values were computed using Chi-square and Fisher’s exact test wherever appropriate. *P* values in bold are those found to be statistically significant.

### Drug susceptibility profile of ETR, RPV and DOR according to drug resistance interpretation algorithms

#### Etravirine

High-level resistance to ETR was found predominantly with the ANRS algorithm (38.0%), while the Stanford HIVdb algorithm gave the highest rate of intermediate resistance (37.5%). The most predominant high-level RAM was Y181C (23.74%), followed by K101E (8.42%) and L100I (5.81%) (Table 2). Among intermediate-level RAMs, V90I was the most predominant (22.81%), followed by A98G (18.83%), E138A (4.13%), M230L (4.28%), G190A (18.68%) and V106I (2.60%). In terms of drug susceptibility, 47.17% [43.37–51.00] of individuals harboured a virus found to be fully susceptible to ETR according to the HIVdb algorithm (Table 3).

**Table 3.** Susceptibility of 2ndGenNNRTI among individuals failing ARV therapy according to drug resistance interpretation algorithms

Drug resistance interpretation algorithms			
Resistance level	Stanford HIVdb	ANRS	HIV-GRADE
Etravirine			
Susceptible	308 (47.2)	301 (46.1)	317 (48.5)
Intermediate resistance	245 (37.5)	104 (15.9)	197 (30.2)
High resistance	100 (15.3)	248 (38.0)	139 (21.3)
Rilpivirine			
Susceptible	210 (32.2)	236 (36.1)	294 (45.0)
Intermediate resistance	167 (25.6)	43 (6.6)	114 (17.5)
High resistance	276 (42.3)	374 (57.3)	245 (37.5)
Doravirine			
Susceptible	216 (33.1)	276 (42.3)	331 (50.7)
Intermediate resistance	267 (40.9)	82 (12.5)	121 (18.5)
High resistance	170 (26.0)	295 (45.2)	201 (30.8)

For the Stanford HIVdb algorithm, resistance to NNRTIs was interpreted using the genotypic scoring system for drug susceptibility with the following penalty: ≥60 high resistance; 15–59: intermediate resistance and <15: susceptible.

#### Rilpivirine

More than 57% of individuals with high-level resistance to RPV were detected with the ANRS algorithm, while HIVdb and HIV-GRADE had 42.3% and 37.5%, respectively (*P* < 0.001). The most common major RAM was Y181CIV (25.11%), followed by K101P (1.83%), Y188L (8.42%), H221Y (8.42%), L100I (5.81%), M230L (4.28%), F227C (4.28%) and E138AGKQ (8.57%) (Table 2). In terms of drug susceptibility, a total of 32.16% [28.69–35.84] of individuals harboured a virus found to be fully susceptible to RPV according to the HIVdb algorithm (Table 3). This proportion was higher by using ANRS (36.1%) and HIV-GRADE (45.0%) algorithms (*P* < 0.001), although this remained below 50% of the total of patients analysed.

#### Doravirine

The rate of high-level resistance to DOR was predominantly found with the ANRS algorithm (45.2%) versus 26% with the HIVdb algorithm. The intermediate-level resistance was found to be higher with the HIVdb algorithm (40.9%), and only 12.6% and 18.5% with ANRS and HIV-GRADE, respectively. In the high-level resistance group, Y188L was the most prevalent RAM (8.42%), followed by F227L (4.28%), M230L (24.28%) and Y318F (0.31%) (Table 2). Mutation P225H was the most predominant intermediate-level resistance mutation (14.70%), followed by L234I (2.30%). In terms of drug susceptibility, 33.08% [29.58–36.78] of individuals harboured a virus fully susceptible to DOR according to the Stanford algorithm (Table 3).

### Association between second-generation NNRTIs resistance and demographic, treatment and virological parameters

**Figure 2.**
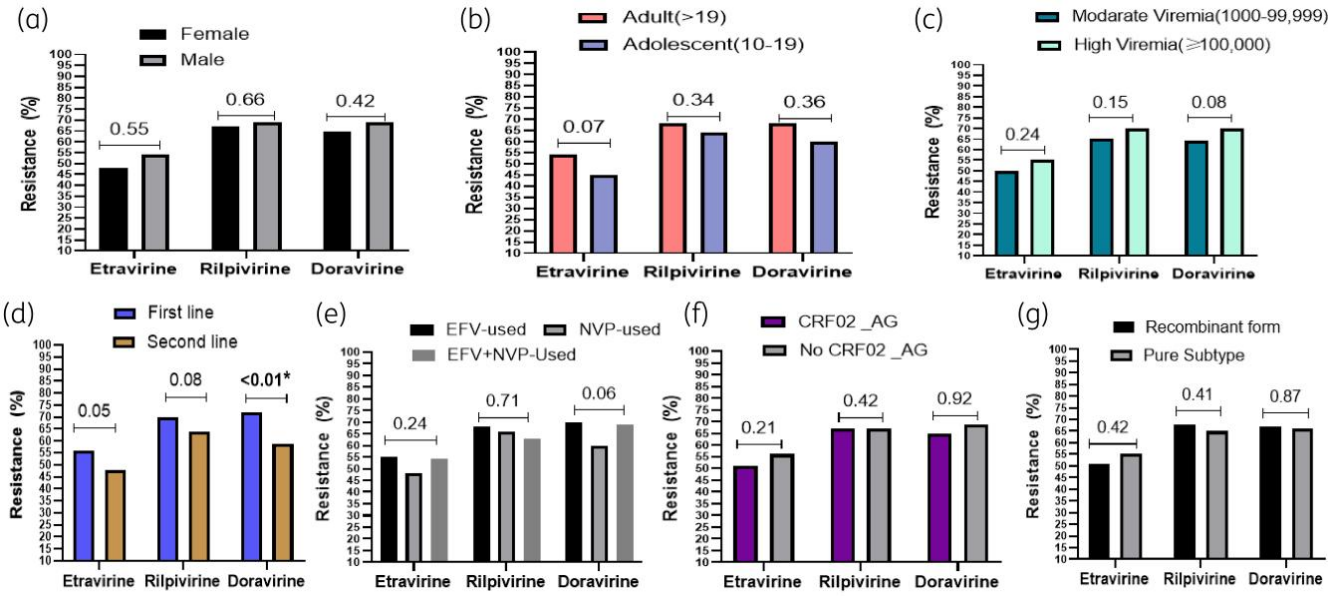
Association between 2ndGenNNRTI resistance and sex, ART lines and virological parameters. (a) Resistance rate according to gender. (b) Resistance rate according to age groups. (c) Resistance rate according to viraemia levels. (d) Resistance rate according to ART lines. (e) Resistance rate according to exposure to 1stGenNNRTIs. (f) Resistance rate according to CRF02_AG versus non CRF02_AG. (g) Resistance according to pure subtypes versus recombinant subtypes. *significant with *P* < 0.05.

According to sex, age groups and viraemia levels, no significant difference was found in the resistance rates of ETR, RPV and DOR (Figure 2a–c). According to ART line, we found a significant difference only in the resistance rate to DOR between first-line (72.85%) and second-line treatment (59.58%), *P* < 0.001 (Figure 2d). On the other hand, even though the individuals exposed to EFV had a higher rate among the three 2ndGenNNRTIs (ETR 55%, RPV 68% and DOR 70%), no significant difference was found when compared to individuals exposed to NVP or both NVP and EFV (Figure 2). According to HIV-1 diversity, we did not see any significant difference between pure subtypes and recombinant forms (Figure 2g).

### Concordance between HIVdb, ANRS and HIV-GRADE algorithms in predicting resistance to second-generation NNRTIs

Regarding ETR susceptibility, there was a positive and strong correlation between HIVdb database and ANRS algorithms (*K*_t_ = 0.68, *P* < 0.001). Likewise, a strong correlation was observed between HIVdb and HIV-GRADE (*K*_t_ = 0.79, *P* < 0.001) (Table S1, available as Supplementary data at *JAC-AMR* Online).

Concerning RPV susceptibility, Kendall’s tau rank correlation test was very strong between the three algorithms. In particular, Kendall’s tau between Stanford and ANRS was 0.83 (*P* < 0.001); and 0.75 (*P* < 0.001) between HIVdb and HIV-GRADE (Table S2).

For DOR susceptibility, the correlation was strong between HIVdb and ANRS (0.72, *P* < 0.001), while a slightly lower correlation was observed between HIVdb and HIV-GRADE (0.60, *P* < 0.001) (Table S3).

## Discussion

In this study among PLHIV failing ART with a focus on cross-resistance between 1st- and 2ndGenNNRTIs, findings to support considerations for the future use of 2ndNNRTI-based regimens, especially those in LA regimens in Cameroon and similar LMICs, have been outlined. In general, the rate of NNRTI resistance mutations was high, with >90% of individuals harbouring RAMs to NNRTIs. This is consistent with the fact that NNRTIs have been extensively used in Cameroon and they have a low genetic barrier to resistance.^32^ Overall, our analyses found a high rate (about 77.18%) of resistance mutations to one or more 2ndGenNNRTI among individuals with exposure to 1stGenNNRTIs failing an NNRTI- or PI-based regimen. As expected, a higher rate was especially found among those failing their first-line NNRTI-based regimens when compared to second-line PI-based regimens.

RAMs were detected in most of the position with reported common cross-resistance with 2ndGenNNRTIs such as L100, K101, Y181, Y188 and M230. Concerning 2ndGenNNRTI RAMs with high-level resistance, Y181C was the most prevalent in our studies, similarly to some other mutations such as K103N and Y181C that frequently emerge in individuals failing 1stGenNNRTIs.^12,33^ Other frequent RAMs to 2ndGenNNRTIs in this study were K101E and Y188L. Several mutations with low-level resistance to 2ndGenNNRTIs such as A98G, E138A/G/Q, G190E, H221Y and P225H were also found.^32,34^ It was interesting to find a considerable number of RAMs to 2ndGenNNRTIs (71.57%) in individuals failing a second-line ART, even though they had a previous exposure to EFV and/or NVP. Indeed, viruses with NNRTI resistance mutations conserve a high replicative capacity in individuals who have failed ART and can therefore persist for years in the plasma.^35^

Overall, only 10.56% of the participants did not harbour a virus with NNRTI-RAMs and were thus fully susceptible to all 2ndGenNNRTI (Table 2). The susceptibility to individual 2ndGenNNRTIs varied between 46.1% and 48.5% for ETR, 32.2% and 45.0% for RPV, and 33.1% and 50.7% for DOR, depending on the tool used for DRM interpretation (Table 3). The observed variation was due to the difference in the interpretation of some specific drug resistance mutations by each algorithm. It therefore appears that the 2ndGenNNRTIs with a high resistance rate in the Cameroonian context is RPV. Of note, the susceptibility was significantly lower when using the Stanford algorithm for both RPV and DOR. A study by Sungkanuparph *et al*. showed that, ETR susceptibility was observed in 26% of individuals, which is lower than the proportion found in our reports.^36^ Also, a study in Ethiopia showed that, among individuals failing first-line ART, RPV susceptibility was 45% using the Stanford algorithm,^37^ which was higher compared to the data found in the present study. Similarly, the susceptibility to DOR found in this study was lower that of a report from Botswana (80.19%).^11^ This discrepancy is probably linked to differences in the characteristics of study population, type of treatment (fewer been exposed to NNRTIs in Botswana), duration of the failing therapy and the fact that different versions of the same algorithm (following updates between the two studies) could have different weights for the same mutation. However, our results are generally similar to several datasets from other LMICs.^38,39^

Looking at the association between 2ndGenNNRTIs and individuals’ characteristics, we did not observe any significant difference in terms of sex, age and viraemia level for all three drugs, suggesting that these characteristics might not be useful in defining the profile of PLHIV that will benefit from 2ndGenNNRTIs in such LMICs. Likewise, no significant difference was found according to whether the individuals were previously exposed to only NVP, EFV or both NVP and EFV; this can be justified by the fact that both EFV and NVP are low genetic barrier drugs, with similar resistance profile.^28,32^ It was also interesting to observe that, susceptibility to 2ndGenNNRTIs did not depend on whether the individuals harboured HIV-1 pure or recombinant strains; this observation is meaningful in a context such as Cameroon and even at the global level where HIV-1 genetic diversity could be concerning.^40–42^ Even though resistance rate to DOR was high among individuals failing second-line regimens (59%) in our study, the burden was significantly lower when compared to those failing first-line regimens (72%), *P* < 0.05. In fact, this observation was expected because all the individuals on second-line regimen were previously exposed to 1stGenNNRTIs. In effect, the lower rate of resistance observed in the second-line group may also be due to archiving of RAMs in cellular reservoirs or within minority viral populations considering the gap of time between ART failure under NNRTI-based regimen before switching to PI-based protocols.^43,44^ In addition, DOR has a different genotypic resistance pathway from other NNRTIs.^45^ Thus, patients failing 1stGenNNRTI should be spared from 2ndGenNNRTI. This is of paramount importance from the clinical point of view, since at the moment resistance testing is not widely available in LMICs and a result generated with Sanger does not preclude the absence of RAMs. This calls for prioritizing 2ndGenNNRTI only for beneficiaries without previous exposure to NNRTIs.

Consensus agreement of algorithms used to predict the HIVDR, including the IAS list, HIVdb, ANRS algorithm and HIV-GRADE, might be an efficient approach to determine the potential efficacy of drugs. Our findings revealed a good agreement between these three algorithms (HIVdb, ANRS and HIV-GRADE). This indicates an overall interoperability between these existing systems in place. However, concerning RPV and DOR, the proportion of susceptible individuals was significantly lower using the HIVdb algorithm. The moderate concordance observed between HIVdb with ANRS and HIV-GRADE for DOR-resistance analysis was probably due to mutations Y318F (considered a major NNRTI-RAM by HIVdb and minor NNRTI-RAM by HIV-GRADE) and F227L (considered a major NNRTI-RAM by HIVdb and mutations associated with possible resistance to NNRTI by ANRS and HIV-GRADE). For ETR resistance analysis, the moderate concordance (*K*_a_ = 0.43) observed between the Stanford database with ARNS is probably due to mutation L100I (considered a major NNRTI-RAM by the Stanford database and mutations associated with possible resistance to NNRTI by ANRS). Concerning RPV, the difference is probably due to mutations H221Yand F227L (considered an intermediate NNRTI-RAM by the Stanford database and minor NNRTI-RAM by HIV-GRADE). All these observations might imply that, using an algorithm with more stringent rules such as the HIVdb might be preferable when considering the use of LA regimen such as CAB + RPV. This is because, this two-drug combination can only be used in the absence of resistance mutations to CAB and RPV. However, in LMICs such as Cameroon where historical exposure to first-line NNRTI is wide, access to resistance testing remains limited and RPV cross-resistance is high, CAB + RPV regimen may not be a safe choice for individuals who previously failed a regimen based on NVP or EFV.^22^ The successful implementation of LA acting regimens containing 2ndGenNNRTIs requires overcoming the following study limitations. Inaccessibility to the time of switch from NNRTI to second-line PI/r could have further explained the lower prevalence of NNRTI mutations and better shape decision-making. Furthermore, CAB + RPV could be advantageous for those switching from first-line dolutegravir-based ART (preferably based on surveys of INSTI-RAMs) to confirm the preserved efficacy of CAB from risks of cross-resistance with DTG. Importantly, studies focused on resistance testing using next generation sequencing will depict the efficacy of LA INSTI + 2ndGenNNRTIs in LMICs.

### Conclusion

In this LMIC with a wide range of HIV-1 genetic diversity and a long-term exposure to 1stGenNNRTI at population-level, there is an alarming rate of NNRTI-RAMs, with a high-level of cross-resistance to 2ndGenNNRTI among patients failing ART. This underscores that, in the absence of a personalized approach (based mostly on resistance testing), the use of any NNRTI-sparing LA regimens should be recommended at this point for public health approach. The rate of RAM to 2ndGenNNRTI among individuals failing their first- or second-line ART was alarming, with low proportions of individuals fully susceptible to ETR, RPV and DOR. Regarding resistance interpretation algorithms, there is a good agreement between HIVdb, ANRS and HIV-GRADE algorithms in predicting susceptibility to second 2ndGenNNRTIs. This supports interoperability or the use of such combination approach to screen for patients before the use of 2ndGenNNRTI-containing rilpivirine LA in Cameroon and in several LMICs sharing similar programmatic features.

## Supplementary Material

dlaf059_Supplementary_Data

## Data Availability

HIV-1 RT sequences generated in this study are available in GenBank under the following accession numbers: OQ985493–OQ985958; OR259435–OR259523; OR259542–OR259566; OR259577–OR259599; OR259764–OR259779; OR259787–OR259819 and OR365155.

## Supplementary data

Tables S1 to S3 are available as Supplementary data at *JAC-AMR* Online.
