# Supplementary material for: HIV-1 cross-resistance to second-generation non-nucleoside reverse transcriptase inhibitors among individuals failing antiretroviral therapy in Cameroon: implications for the use of long-acting treatment regimens in low- and middle-income countries
Source: JAC Antimicrob Resist. 2025 Apr 28;7(2):dlaf059. doi: 10.1093/jacamr/dlaf059 (PMC12034458; doi:10.1093/jacamr/dlaf059)
Supplement: dlaf059_Supplementary_Data [file dlaf059_supplementary_data.docx]

**Supplementary Table 1:** Concordance between the ***HIVdb*** with ANRS, and HIV-GRADE algorithms in predicting resistance to ETR

| 1. ***HIVdb* Versus ANRS** | | | | | |
| --- | --- | --- | --- | --- | --- |
|  |  | ***ANRS*** | | |  |
|  |  | Susceptible | Intermediate resistance | High-resistance | Total |
| ***HIVdb*** | Susceptible | 255 | 37 | 16 | 308 |
|  | Intermediate resistance | 41 | 62 | 142 | 245 |
|  | High-resistance | 5 | 5 | 90 | 100 |
| Total | | 301 | 104 | 248 | 653 |
| (Kendall's tau= 0.68; p<0.001);  (K(Kappa)=0.43, p<0.001) | | | | | |
| 1. ***HIVdb* Vs HIV-GRADE** | | | | |  |
|  |  | HIV-GRADE | | |  |
| HIVdb |  | Susceptible | Intermediate resistance | High-resistance | Total |
|  | Susceptible | 284 | 17 | 7 | 308 |
|  | Intermediate resistance | 29 | 169 | 47 | 245 |
|  | High-resistance | 4 | 11 | 85 | 100 |
|  | Total | 317 | 197 | 139 | 653 |
| (Kendall's tau= 0.79; p<0.001) ;  (K(Kappa)=0.72, p<0.001) | | | | | |

*Legend:* *For HIVdb algorithm, resistance to NNRTIs was interpreted using the genotypic scoring system for drug susceptibility with the following penalty: ≥60 high-resistance; 15–59: intermediate-resistance; <15: susceptible.*

**Supplementary Table 2:** Concordance between Stanford with ANRS, and HIV-GRADE algorithms in predicting resistance to RPV

| 1. ***HIVdb* Versus ANRS** | | | | | |
| --- | --- | --- | --- | --- | --- |
| **ANRS** | | | | | |
|  |  | Susceptible | Intermediate resistance | High-resistance | Total |
| **HIVdb** | Susceptible | 210 | 0 | 0 | 210 |
|  | Intermediate resistance | 24 | 42 | 101 | 167 |
|  | High-resistance | 2 | 1 | 273 | 276 |
| Total | | 236 | 43 | 374 | 653 |
| (Kendall's tau= 0.83; p<0.001); (K(Kappa)=0.69, p<0.001) | | | | | |
| ***HIVdb* Versus HIV-GRADE** | | | | | |
| **HIV-GRADE** | | | | | |
|  |  | Susceptible | Intermediate resistance | High-resistance |  |
| **HIVdb** | Susceptible | 204 | 1 | 5 | 210 |
|  | Intermediate resistance | 79 | 60 | 28 | 167 |
|  | High-resistance | 11 | 53 | 212 | 276 |
| Total |  | 294 | 114 | 245 | 653 |
| (Kendall's tau= 0.75; p<0.001); (K(Kappa)=0.59, p<0.001) | | | | | |

*Legend:* *For Stanford HIVdb algorithm, resistance to NNRTIs was interpreted using the genotypic scoring system for drug susceptibility with the following penalty: ≥60 high-resistance; 15–59: intermediate-resistance; <15: susceptible.*

**Supplementary Table 3**: Concordance between HIVdb with ANRS, and HIV-GRADE algorithms in predicting resistance to DOR

| 1. ***HIVdb* Versus ANRS** | | | | | |
| --- | --- | --- | --- | --- | --- |
|  |  | ***ANRS*** | | |  |
|  |  | Susceptible | Intermediate resistance | High-resistance | Total |
| ***HIVdb*** | Susceptible | 213 | 3 | 0 | 216 |
|  | Intermediate resistance | 54 | 69 | 144 | 267 |
|  | High-resistance | 9 | 10 | 151 | 170 |
| Total | | 276 | 82 | 295 | 653 |
| (Kendall's tau= 0.72; p<0.001) ;  (K(Kappa)=0.51, p<0.001) | | | | | |
| 1. ***HIVdb* Versus HIV-GRADE** | | | | |  |
|  |  | ***HIV-GRADE*** | | |  |
|  |  | Susceptible | Intermediate resistance | High-resistance |  |
| ***HIVdb*** | Susceptible | 194 | 16 | 6 | 216 |
|  | Intermediate resistance | 119 | 79 | 69 | 267 |
|  | High-resistance | 18 | 26 | 126 | 170 |
|  | Total | 331 | 121 | 201 | 653 |
| (Kendall's tau= 0.60 ; p<0.001) ;  (K(Kappa)=0.56, p<0.001) | | | | | |

*Legend:* *For HIVdb algorithm, resistance to NNRTIs was interpreted using the genotypic scoring system for drug susceptibility with the following penalty: ≥60 high-resistance; 15–59: intermediate-resistance; <15: susceptible.*
